# Profile of metacaspase gene expression in *Plasmodium vivax* field isolates from the Brazilian Amazon

**DOI:** 10.1007/s11033-024-09538-x

**Published:** 2024-04-29

**Authors:** Carolina Moreira Blanco, Hugo Amorim dos Santos de Souza, Priscilla da Costa Martins, Camila Fabbri, Fernanda Souza de Souza, Josué da Costa Lima-Junior, Stefanie Costa Pinto Lopes, Lilian Rose Pratt-Riccio, Cláudio Tadeu Daniel-Ribeiro, Paulo Renato Rivas Totino

**Affiliations:** 1 Laboratório de Pesquisa em Malária, Instituto Oswaldo Cruz, Fiocruz and Centro de Pesquisa, Diagnóstico e Treinamento em Malária (CPD-Mal), Secretaria de Vigilância em Saúde e Ambiente (SVSA), Ministério da Saúde, Rio de Janeiro, Brasil; 2 Instituto Leônidas e Maria Deane, Fiocruz Amazônia, Manaus, Brasil; 3 Fundação de Medicina Tropical Dr. Heitor Vieira Dourado (FMT-HVD), Manaus, Brasil; 4 Laboratório de Imunoparasitologia, Instituto Oswaldo Cruz, Fiocruz, Rio de Janeiro, Brasil

**Keywords:** Malaria, Drug target, *P. vivax*, Metacaspases

## Abstract

**Background:**

Metacaspases comprise a family of cysteine proteases implicated in both cell death and cell differentiation of protists that has been considered a potential drug target for protozoan parasites. However, the biology of metacaspases in *Plasmodium vivax* − the second most prevalent and most widespread human malaria parasite worldwide, whose occurrence of chemoresistance has been reported in many endemic countries, remains largely unexplored. Therefore, the present study aimed to address, for the first time, the expression pattern of metacaspases in *P. vivax* parasites.

**Methods and results:**

*P. vivax* blood-stage parasites were obtained from malaria patients in the Brazilian Amazon and the expression of the three putative *P. vivax* metacaspases (*Pv*MCA1-3) was detected in all isolates by quantitative PCR assay. Of note, the expression levels of each *Pv*MCA varied noticeably across isolates, which presented different frequencies of parasite forms, supporting that *Pv*MCAs may be expressed in a stage-specific manner as previously shown in *P. falciparum*.

**Conclusion:**

The detection of metacaspases in *P. vivax* blood-stage parasites reported herein, allows the inclusion of these proteases as a potential candidate drug target for vivax malaria, while further investigations are still required to evaluate the activity, role and essentiality of metacaspases in *P. vivax* biology.

## Introduction

Metacaspases are cysteine proteases belonging to the C14B subfamily of peptidases that present structural homology to metazoan caspases [1] − the well-known components of programmed cell death pathways in mammalian cells, which also play a role in non-death related processes [2]. Absent in metazoa, metacaspases are found in the genome of prokaryotes, protists, fungi, and plants and, since their first description by Uren and colleagues in 2000 [3], metacaspases have been implicated in a variety of functions besides cell death, including regulation of proteostasis in yeasts and defense against pathogens in plants [4, 5]. In protozoans, metacaspases also seem to be involved in cell differentiation and proliferation, being considered potential drug targets [6, 7].

In the genus *Plasmodium*, which comprises the causative agents of malaria, three metacaspases (MCA1-3) were previously identified by comparative sequence analysis [8, 9] and studies on their expression and activity are limited to the rodent parasite *P. berghei* as well as to *P. falciparum* − the most prevalent and deadly human malaria parasite worldwide [10–13]. The frequent emergence of chemoresistance in *P. falciparum* parasites certainly propelled the knowledge of *P. falciparum* metacaspases (*Pf*MCAs), while *P. vivax* MCAs (*Pv*MCAs) have been neglected, despite *P. vivax* impacting significantly on public health in many malaria endemic countries outside of sub-Saharan Africa, where antimalarial drug resistance is also found in *P. vivax* infections [14, 15]. Although there are published work on *Pv*MCAs, all of them are focused on genetic diversity of *Pv*MCA1 [16–18] and no study of MCA expression has been published.

## Methodology

To examine if the putative genes for *Pv*MCAs are expressed, blood-stage forms of *P. vivax* were obtained from malaria patients attended to at the *Fundação de Medicina Tropical Doutor Heitor Vieira Dourado* (*FMT-HVD*) in Manaus, Brazil, according the procedures approved by the Research Ethics Committee of FMT-HVD (CAAE  75894223.9.0000.0005). Diagnosis was done by Giemsa-stained thick blood smears examination and, then, peripheral blood heparinized samples were collected from four patients presenting parasitemia higher than 500 parasites/µl. Subsequently, parasites were concentrated by 70% Percoll density gradient centrifugation (GE-Healthcare) after depletion of leukocytes in cellulose columns (Sigma), as described elsewhere [19], and differential frequency of each parasite form (rings, trophozoites, schizonts and gametocytes) in the concentrated parasite samples was estimated after counting at least 1,000 erythrocytes in thin smears stained with Giemsa. Lastly, total number of enriched parasites per sample was determined in a Neubauer chamber.

The total RNA was extracted from ≥ 1 × 10^7^ enriched parasites using PureLink RNA mini-Kit (Ambion), followed by treatment with DNase (Invitrogen) and reverse transcription using the high-capacity cDNA reverse transcription kit (Applied Biosystems). Real-time quantitative PCR (qPCR) assays were carried out in duplicate using a 7500 Real-Time PCR System (Applied Biosystems) with 20 µL reaction solution containing 10 ng cDNA, 1X PowerUp SYBR Green Master Mix (Applied Biosystems), 600 nM of forward and reverse primers (GENONE), and UltraPureDNase/RNase-Free Distilled Water (Invitrogen). Thermocycling conditions used were as follows: 2 min at 50 °C, followed by 2 min at 95 °C and 40 cycles of denaturation (95 °C/15 s) and annealing (60 °C/1 min). After the last cycle, a melting curve was performed (95 °C/15 s, 60 °C/1 min, 95 °C/15 s) to check the specificity of amplification.

Primers for *Pv*MCA1 amplification were selected from Sow et al., 2017 [17] and primers for *Pv*MCA2 and 3 (Table 1 ) were designed according to the sequences of the genes available in the PlasmoDB database (*Pv*MCA1: PVX_114725; *Pv*MCA2: PVX_118575; *Pv*MCA3: PVX_085640) using Primer-Blast [20] and PCR Primer Stats [21]. β-tubulin and 18 S rRNA housekeeping genes of *P. vivax* were used as internal controls [22, 23] (Table 1), and relative gene expression was calculated using the 2^−ΔΔCt^ method [24].

**Table 1:** Primer sequences used in gene expression assays of *P. vivax* metacaspases (*Pv*MCAs) by qPCR

Gene	Prime sequence		Product size (bp)
	Forward (5′–3′)	Reverse (5′–3′)	
*Pv*MCA1	ACCCCAGTGGACCACCAA	CACGAGGGTAAGTAACCCCA	110
*Pv*MCA2	ACACCCTGGAAATGTGCGAA	AGCCTTTTGAGCGACGAAGT	107
*Pv*MCA3	TGTTCCGACCCCTTTAACCG	ATGGTTTGACAGCCTGAGCA	131
18 S rRNA	TTTCTCTTCGGAGTTTATTCTTAGATT	GTCAAATTAAGCCGCAAGCT	154
β-tubulin	CCAAGAATATGATGTGTGCAAGTG	GGCGCAGGCGGTTAGG	59

## Results and discussion

Metacaspases have been widely studied in plants and differential patterns of expression are observed among them as well as among different tissues of various species studied, in which up to nine metacaspases have been described [25–27]. In protozoa, although a variable number of metacaspases are found across the different taxa and the putative roles of these proteases have been described [4, 7], a comparative analysis of the expression levels among the metacaspases from a given species has not yet been done. Therefore, herein, to investigate the expression profile of the three metacaspases of *P. vivax*, for which a continuous *in vitro* culture is not yet available, blood-stage parasites were obtained from four malaria vivax patients.

**Fig. 1:**
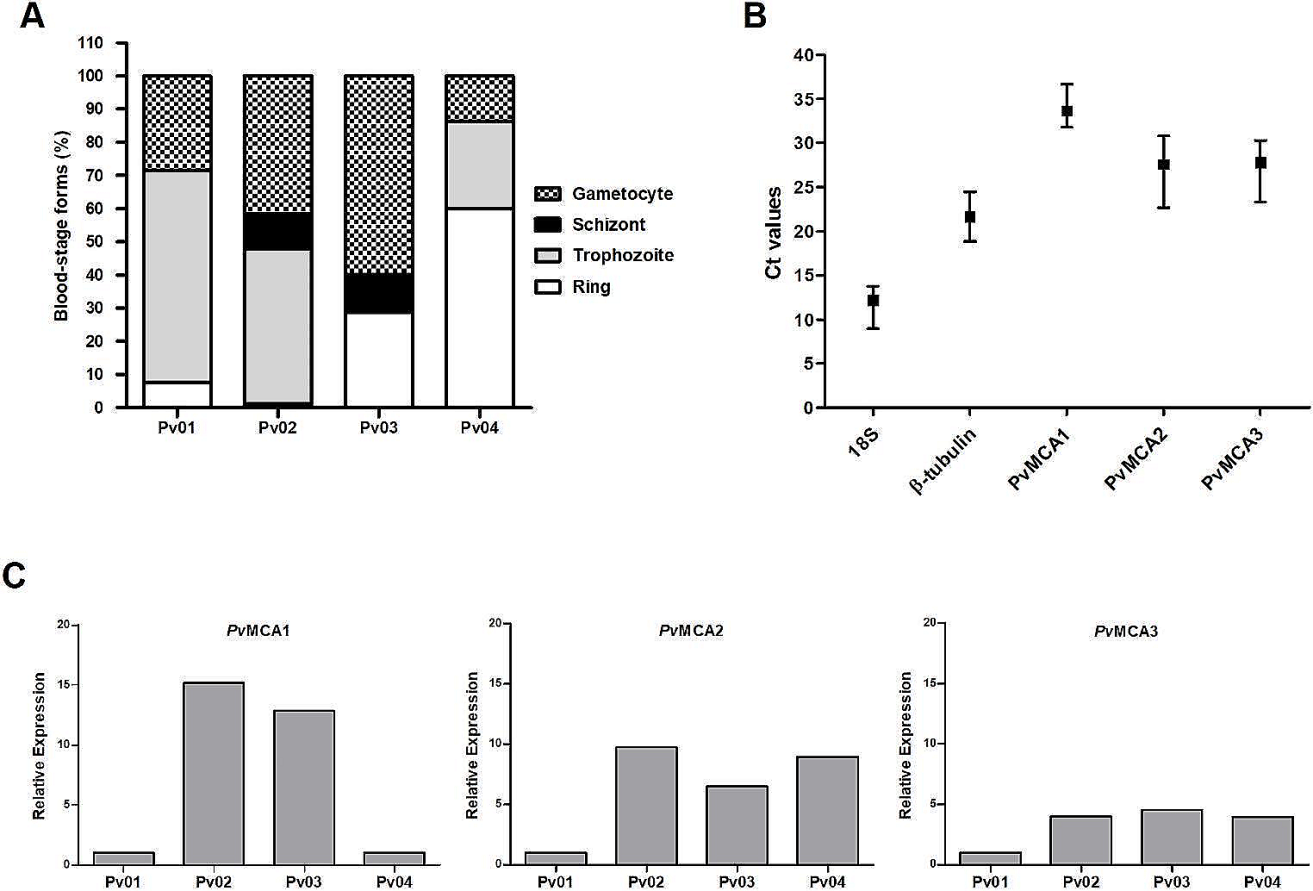
Profile of metacaspase expression in *P. vivax* blood-stage parasites isolated from malaria patients. **A** Frequency of blood-stage forms in *P. vivax* samples (Pv01-04) after parasite enrichment by 70% Percoll centrifugation. **B** Mean threshold cycle (Ct) values for *P. vivax* metacaspases (*Pv*MCA1, *Pv*MCA2 and *Pv*MCA3) and *P. vivax* housekeeping genes (18 S rRNA and ß-tubulin), as evaluated by real-time quantitative PCR (qPCR) in *P. vivax* isolates (Pv01-04). The bars indicate the maximum and minimum Ct values detected, respectively. **C** Relative expression of *Pv*MCA1, *Pv*MCA2 and *Pv*MCA3 among *P. vivax* isolates. The 18 S rRNA gene was used as internal control and Pv01 was selected as calibrator sample for ∆∆Ct calculation. Data are expressed as 2^−∆∆Ct^ values

Parasite samples were initially enriched by Percoll gradient centrifugation and, as shown in Fig. 1A, presented variable frequencies of blood-stage forms, including rings, trophozoites, schizonts, and gametocytes. Expression of metacaspase genes was, then, evaluated by qPCR using 18 S and ß-tubulin genes as internal control and, in this manner, it was possible to detect the expression of the three predicted genes for *Pv*MCAs (*Pv*MCA1, *Pv*MCA2, and *Pv*MCA3) in all samples examined (Fig. 1B). According to Ct (threshold cycle) values, genes for *Pv*MCAs presented low levels of expression compared to both housekeeping genes assayed; with *Pv*MCA1 showing the higher Ct values (Ct variation: 31.78 to 36.67), followed by *Pv*MCA2 and *Pv*MCA3, whose profile of expression were quite similar (Ct variation: 22.67 to 30.84 and 23.32 to 30.31, respectively). Overall, variation of Ct values was not very discrepant across genes examined and the lowest and highest variations were exhibited by 18 S and *Pv*MCA2, which varied 4.81 and 8.17 cycles, respectively (Fig. 1B).

The detection of the *Pv*MCAs observed in the blood-stages of *P. vivax* agrees with previously published results on *P. falciparum* and *P. berghei* [8, 11–13]. Taken together, these studies demonstrate that all three *Plasmodium* metacaspases (MCA1-3) are expressed in blood-stages of *P. falciparum* and, differently from *Pb*MCA2, whose expression levels were uniform over parasite mosquito stages [13], *Pf*MCA2 and *Pf*MCA3 showed a stage specific pattern [11, 12], while data on *Pf*MCA1 expression were restricted to asynchronous culture [8]. Indeed, additional relative expression analysis for each gene revealed that *Pv*MCAs were not equally expressed among analysed parasite samples (Fig. 1C), which is possibly a result of the variable quantity of each blood-stage form present in the samples (Fig. 1A). In *P. falciparum*, for instance, *Pf*MCA2 was detectable in schizonts and gametocytes, whereas *Pf*MCA3 expression was higher in rings and schizonts [11, 12]. However, excepting *Pv*MCA1 that was markedly increased in the two samples containing both schizonts and the highest percentages of gametocytes (Pv02 and Pv03), no clear expression pattern relative to the frequency of parasite forms was noticed for *Pv*MCA2 and *Pv*MCA3 (Fig. 1A and C). Alternatively, the variation in the expression of each metacaspase gene observed among the *P. vivax* isolates (Fig. 1C) could be a result of the populational heterogeneity of the parasites that occurs in endemic areas, as previously shown for *P. falciparum* and *P. vivax* genes related to erythrocyte invasion or chemoresistance [28, 29]. Doubtlessly, further studies employing individually purified parasite forms obtained from different isolates may help to determine the stage-specific expression of the *Pv*MCAs.

Even though our data demonstrate that the metacaspases genes are expressed in *P. vivax*, the role of them is still unknown and the elucidation of their essentiality for the parasite biology is impaired by the absence of a continuous *in vitro* culture for *P. vivax*. Nevertheless, genome-scale mutagenesis screen in *P. falciparum* identified *Pf*MCA3 gene as essential for the asexual blood-stage [30] and, more recently, a marked involvement of *Pb*MCA2 in the sexual stage development was shown using *P. berghei* knockout parasites [13], supporting a pivotal participation of metacaspases in different phases of *Plasmodium* life cycle. In this context, studies focusing the metacaspases of the simian malaria parasite *P. cynomolgi*, which has been proposed as a model system for *in vivo* and *in vitro* research on *P. vivax* [31, 32], could bring some light into the functionality of *Plasmodium* metacaspases, especially the *Pv*MCAs.

In conclusion, it is shown for the first time that the three metacaspases described in the genus *Plasmodium* (MCA1-3) are expressed in the blood-stage forms of *P. vivax* at least at the transcriptional level and presumably in a stage-specific manner. Such observations raise the possibility that the metacaspase family can also be a candidate drug target for *P. vivax*, although the essentiality of each *Pv*MCAs for the parasite development is still to be elucidated. Additional studies are currently underway to characterize the protease activity of *Pv*MCAs as well as their involvement in the life cycle of *P. vivax*.

## Data Availability

Data will be made available on request.
